# Allergic contact dermatitis to lettuce^☆^

**DOI:** 10.1016/j.abd.2024.04.006

**Published:** 2024-10-31

**Authors:** Mariany Lima Rezende, Ana Luiza Castro Fernandes Villarinho, Maria das Graças Mota Melo, Clarissa Vita Campos

**Affiliations:** a Hospital Federal de Bonsucesso, Rio de Janeiro, RJ, Brazil; b Centro de Estudos da Saúde do Trabalhador e Ecologia Humana, Escola Nacional de Saúde Pública, Fundação Oswaldo Cruz, Rio de Janeiro, RJ, Brazil

Dear Editor,

Hand contact dermatitis is considered the most common occupational dermatosis (90% to 95% of cases), causing morbidity and functional impairment. The diversity of allergens in the workplace makes it difficult to determine the causative substrate, making the patch test (PT) essential for diagnostic elucidation.^1^

A case of allergic contact dermatitis (ACD) in a food handler triggered by an allergn present in lettuce is described, in which the test with the fresh vegetable was relevant for diagnostic confirmation.

A 46-year-old man, a general services assistant in a fruit and vegetable store, with no history of previous allergies, reported blisters, exudation, and pruritus on his hands for four months. He claimed that the condition worsened when handling green vegetables at work. He was treated with moisturizers, corticosteroids, and topical and systemic antibiotics, without response. On examination, there were erythematous, desquamative, and lichenified plaques, with erosions and fissures on the palms (Fig. 1).

**Figure 1:**
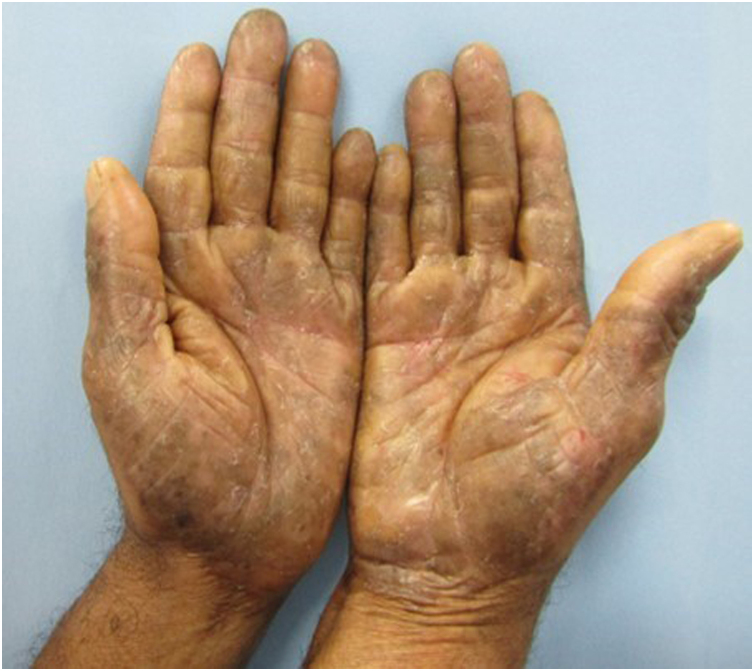
On examination, erythematous, brownish plaques with lichenification, desquamation, erosions and fissures are observed on the hands.

A patch test was performed using a Latin American battery (IPI-ASAC, Brazil), which showed positivity for sesquiterpene lactones (SLs). Due to reports of worsening with the handling of leaves, the CT was also applied using fresh vegetables (cilantro, chives, parsley, watercress, leaves and stems of iceberg and green-leaf lettuce) prepared with a drop of bidistilled water and applied to the skin under occlusion for 48 hours (Fig. 2), as recommended by the International Contact Dermatitis Research Group. The results were positive for both types of lettuce tested at 48 and 96 hours (Fig. 3). The prick-by-prick test with the fresh leaves was negative, ruling out protein contact dermatitis (PCD), another cause of hand eczema.

**Figure 2:**
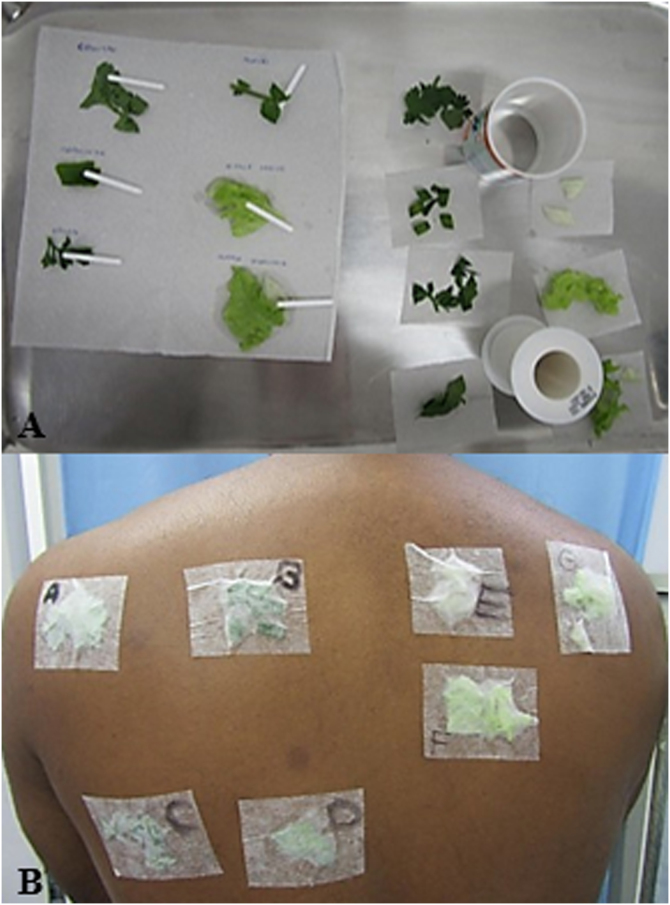
(A) Preparation of leaves with bidistilled water for the prick-by-prick test on the left and contact test on the right. (B) Application of leaves for the contact test identified by letters: (A) Coriander; (B) Chives; (C) Parsley; (D) Watercress; (E) Iceberg lettuce stem; (F) Iceberg lettuce leaf; (G) Leaf and stem of green-leaf lettuce.

**Figure 3:**
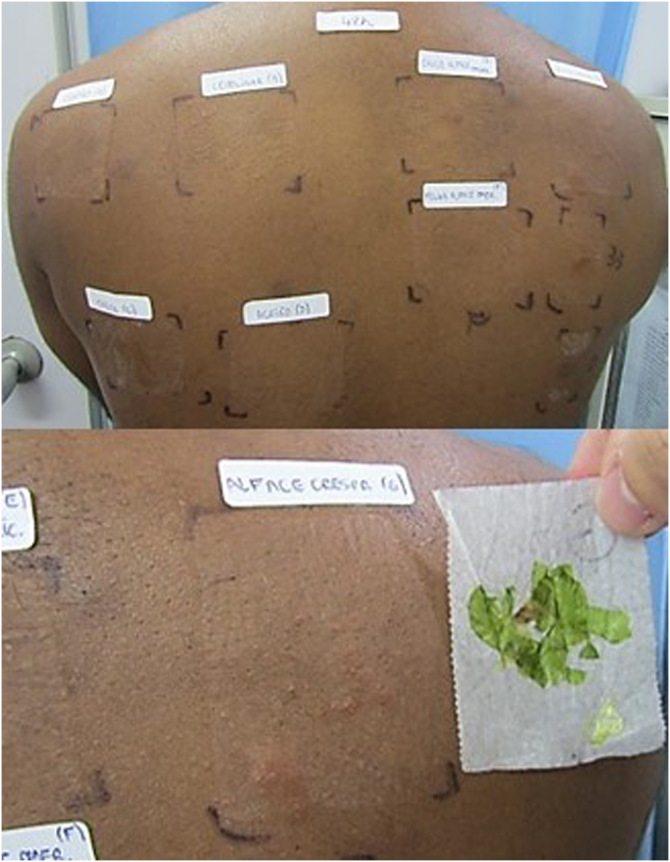
Positive result for green leaf lettuce leaf and stem in the first reading after 48 hours.

The findings corroborated the diagnosis of ACD with an occupational link, with lettuce being the causal agent. The patient was taken off work for thirty days and medicated with an emollient and topical corticosteroid, with lesion resolution. He returned to work, but without direct exposure to vegetables and had no recurrence of the condition (Fig. 4).

**Figure 4:**
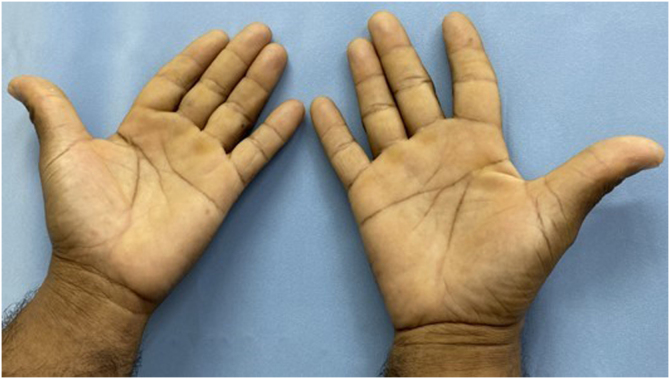
Clinical improvement four months after suspending contact with the allergen, using only moisturizing emollients.

ACD caused by lettuce is rarely reported in the literature, considering its high consumption and cultivation rate. Reports from food handlers prove that anyone involved in the preparation is at risk.^2^

The Latin American battery of patch tests includes forty substances, including SLs, which are metabolites of plants of the *Compositae* family capable of inducing cutaneous cytotoxicity, causing severe dermatitis.^3^ Its sensitivity is tested using a mixture of three compounds in equimolar concentrations: alantolactone (eudesmanolide class), costunolide (germacranolide class) and dehydrocostus lactone (guaianolide class), at a concentration of 0.1% in petrolatum.^4^, ^5^ It rarely causes false-positive reactions, as there is low risk of active sensitization. Cross-reactions may occur with other plants of the *Compositae* family, such as chicory, artichoke, chamomile, sunflowers, and even hygiene and dermo-cosmetic products that contain SLs in their formulation.^2^, ^5^, ^6^

In the case of allergy to lettuce, the test with SLs can be used as a type of screening, since the mix does not contain the two substances that have been proven to be allergenic: lactucin and lactucopicrin.^2^ The diagnosis can be made by patch testing with both at a concentration of 1% in petrolatum, a short ether extract of lettuce at 10% in petrolatum, or the fresh plant and sap. Some authors propose the inclusion of lactucopicrin in a new SL mix (SL mix II).^7^

In the case reported herein, the leaves used in the PT were prepared with double distilled water, but ether can also be used. Dilution is necessary to reduce the caustic effect of the sap, which can cause contact urticaria, leading to false positivity. For research purposes, the authors suggest performing the tests on a control group of patients.^2^

It was decided also to perform the prick-by-prick test to rule out the possibility of PCD, a rarer condition that occurs due to an immediate hypersensitivity reaction caused by high molecular weight proteins that are unable to penetrate intact skin, which is why a prick test is necessary for such diagnosis.^8^

Adequate response to treatment depends on the elimination of the allergen. The case described herein illustrates this statement because despite the previously adopted measures, the dermatitis only resolved after the exposure to lettuce and other vegetables was discontinued. It is noteworthy that the discovery of the substrate that causes eczema, associated with the identification of other mechanisms that trigger the condition prevents relapses and improves patient quality of life.

## Financial support

None declared.

## Authors' contributions

Mariany Lima Rezende: Design and planning of the study.

Ana Luiza Castro Fernandes Villarinho: Critical review of important intellectual content; intellectual participation in the propaedeutic and/or therapeutic conduct of the studied cases.

Maria das Graças Mota Melo: Effective participation in research orientation.

Clarissa Vita Campos: Critical review of the literature; approval of the final version of the manuscript.

## Conflicts of interest

None declared.
